# O.4.1-5 An online lifestyle intervention consisting of resistance exercise and advice on protein intake improves leg muscle strength and physical functioning in older adults

**DOI:** 10.1093/eurpub/ckad133.170

**Published:** 2023-09-11

**Authors:** Berber Dorhout, Pol Grootswagers, Nick Wezenbeek

**Affiliations:** Wageningen University, The Netherlands; Wageningen University, The Netherlands; CleverMove, The Netherlands; berber.dorhout@wur.nl

## Abstract

**Purpose:**

The lifestyle intervention *ProMuscle,* combining resistance exercise and an increased protein intake, was effective in improving muscle strength, muscle mass and physical functioning in older adults. However, the ageing population is increasing at a fast pace. This leads to growing numbers of older adults suffering from the negative consequences associated with ageing, such as a declined physical functioning. Due to insufficient healthcare professionals, the rapidly growing aging population cannot be personally guided in (preventive) care in the future. Innovative online lifestyle interventions provide opportunities to guide larger numbers of older adults, demanding lower deployment of healthcare professionals. The current study investigates the effects of the online version of *ProMuscle* on muscle strength and physical functioning in healthy older adults.

**Methods:**

A single-arm longitudinal study was conducted in the Netherlands. In the 24-week intervention 19 healthy adults aged 55 and older were included. They conducted online resistance training twice a week during 24 weeks. In addition, older adults were advised on increasing protein intake via two online consultation by a dietitian in the first 12 weeks, and via a 12-week online learning platform in the second 12 weeks. The 1 repetition maximum (1RM) knee extension and repeated chair rise test were performed at baseline, in week 12 and 24. Linear mixed models were used to test differences over time. Adherence to the training sessions was measured as number of trainings watched, divided by number of trainings provided, times 100%.

**Results:**

Mean age of participants at baseline was 69±6 years. The 1RM knee extension and repeated chair rise test improved significantly during the 24-week intervention (15.49 lbs, SE 1.92 and -1.16 seconds, SE 0.22 respectively). Adherence to training sessions was 72%.

**Conclusions:**

The online lifestyle intervention ProMuscle, combining exercise and nutrition, can contribute to improved muscle strength and physical functioning in healthy older adults. Results from this one-armed trial should be interpreted with caution and larger trials are needed. Providing such lifestyle interventions online enables an increased number of older adults to follow the program, providing the opportunity to contribute to the health and independence of the rapidly growing ageing population.

